# The potential of neuromelanin-sensitive MRI in psychiatric disorders

**DOI:** 10.1016/j.nsa.2026.106994

**Published:** 2026-03-02

**Authors:** Eline Neutelings, Elsmarieke van de Giessen, Lieuwe de Haan, Marieke van der Pluijm

**Affiliations:** a Department of Psychiatry, Amsterdam UMC, University of Amsterdam, the Netherlands; b Department of Radiology and Nuclear Medicine, Amsterdam UMC, University of Amsterdam, the Netherlands

**Keywords:** Neuromelanin-sensitive MRI, Psychiatric disorders, Dopaminergic system, Noradrenergic system, Neuroimaging

## Abstract

Psychiatric disorders represent a significant public health concern due to their high prevalence and lifelong substantial impact. Despite advances in understanding their neurobiological underpinnings, the transdiagnostic mechanisms driving psychiatric conditions remain unclear. Neuromelanin-sensitive magnetic resonance imaging (NM-MRI) is a non-invasive technique for investigating the dopaminergic and noradrenergic systems, offering valuable insights into their role in psychiatric disorders. This review explores the potential of NM-MRI in psychiatric research, considering its promise to reveal links between dopaminergic and noradrenergic dysregulation and psychiatric conditions, thereby offering insights in neurobiological mechanisms. We summarize current NM-MRI findings across psychiatric disorders, including psychosis, mood disorders, and anxiety disorders. Across studies, NM-MRI provides converging evidence for alterations in catecholaminergic systems in several psychiatric conditions, particularly schizophrenia, substance use disorders, and depression. However, the evidence base remains uneven, with relatively few studies concerning anxiety disorders, obsessive compulsive disorder, and neurodevelopmental disorders, despite their known associations with dopaminergic and noradrenergic dysfunction. At present, most NM-MRI findings derive from cross-sectional studies using heterogeneous acquisition and analysis approaches, which limits direct comparability and clinical translation. To fully establish NM-MRI as a clinical research tool in psychiatric context, further efforts are needed to standardize protocols and improve specificity and reliability. Moreover, longitudinal studies and cross-diagnostic comparisons are required to determine whether NM-MRI measures can contribute to risk stratification, treatment monitoring, or other clinically applications. Overall, NM-MRI represents a valuable research tool for probing catecholaminergic involvement in psychiatric disorders and may, with further validation, contribute to future biomarker development in mental health research.

## 1 Introduction

Psychiatric disorders represent a significant public health challenge due to their high prevalence and debilitating impact on individuals and society (Scott et al., 2018). These conditions commonly exhibit complex and overlapping clinical features and are associated with substantial cognitive and emotional impairments that disrupt daily functioning (Aslan et al., 2024). Despite advances in understanding its neurobiology, the mechanisms underlying many psychiatric disorders remain unclear. Neuroimaging techniques using magnetic resonance imaging (MRI), including functional and structural MRI, have been instrumental in elucidating the neural correlates of these disorders (Nour et al., 2022). Notable structural abnormalities are prevalent across multiple psychiatric disorders, such as major depressive disorder (MDD), schizophrenia, bipolar disorder, attention-deficit/hyperactivity disorder (ADHD), and obsessive-compulsive disorder (OCD). Shared morphometric alterations involve the hippocampus, prefrontal cortex and distributed cortical networks, although significant individual heterogeneity exists within and between disorders (Cattarinussi et al., 2022; Opel et al., 2020; Segal et al., 2023). Additionally, functional abnormalities have been investigated in psychiatric populations using functional imaging techniques, such as task-based functional MRI (fMRI), to examine abnormal brain function. These methods have also been applied to evaluate the effects of pharmacological interventions aimed at restoring brain function, and to identify early markers of psychiatric risk in young individuals (Nielsen et al., 2023; Pilmeyer et al., 2022; Boccia et al., 2016).

**Fig. 1:**
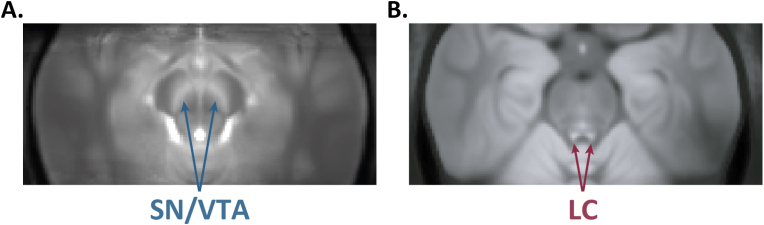
Two examples of NM-MRI images. The images depict the average contrast-to-noise ratio across several individuals in MNI standard space for (A) the substantia nigra/ventral tegmental area (SN/VTA), acquired using a gradient-recalled echo (GRE) sequence with magnetization transfer pulses; and (B) the locus coeruleus (LC), acquired using a GRE sequence with dual-slab saturation pulses.

**Fig. 2:**
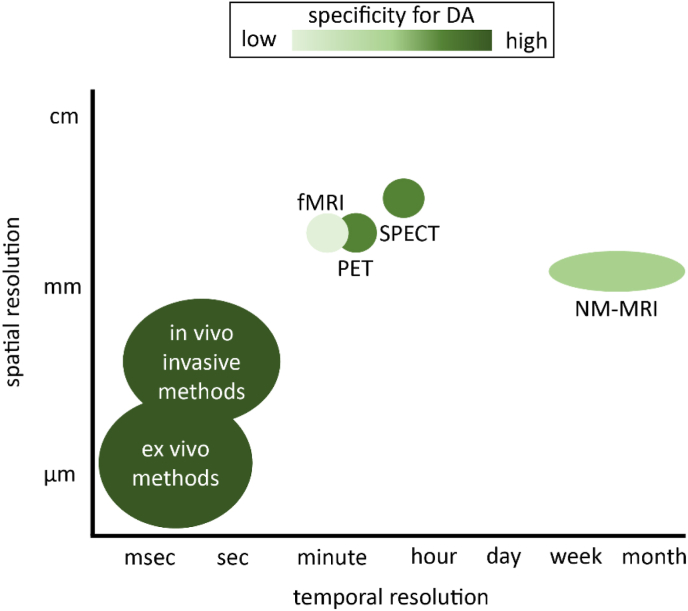
Schematic scale of NM-MRI in comparison to other imaging techniques. NM-MRI has lower spatial and temporal resolution, as well as specificity for dopamine (DA), than invasive in-vivo and ex-vivo methods (e.g. microdialysis, voltammetry, autoradiography), which can only be applied in animal research. Although NM-MRI is less specific for dopamine functioning than certain PET and SPECT tracers, NM-MRI does not use ionizing radiation and has comparable or better spatial resolution. The temporal resolution of NM-MRI is lower due to the nature of neuromelanin as a deposit that accumulates over time, however, advantages of NM-MRI over the other techniques are short scan duration and lower costs. *Figure adapted from Reneman* et al. (Reneman et al., 2021).

Both structural and functional abnormalities may be associated with alterations in catecholaminergic neurotransmission, particularly involving dopamine and noradrenaline (Haber, 2014; Calarco et al., 2022; Goddard et al., 2010; Grace, 2016). Most *in-vivo* assessments of these neurotransmitter systems are conducted using positron emission tomography (PET) or single photon emission computed tomography (SPECT) (Howes et al., 2011; Stone et al., 2009; Moriguchi et al., 2017). These techniques allow quantification of receptor or transporter binding and dynamic neurotransmitter release but involve radiation exposure and are costly, making them unsuitable for routine screening or use in pediatric populations. Dopaminergic neurons are primarily found in the midbrain nuclei, especially in the substantia nigra (SN) pars compacta and the ventral tegmental area (VTA) (Poulin et al., 2018), whereas noradrenergic neurons are mainly concentrated in the locus coeruleus (LC) (Robertson et al., 2013). Disruptions in the SN/VTA and LC systems have been implicated in the onset and progression of various mental health disorders, including schizophrenia (Grace, 2016; Maia and Frank, 2017), depression (Cui et al., 2024), anxiety (Goddard et al., 2010; Gong et al., 2021), post-traumatic stress disorder (Hendrickson and Raskind, 2016), and addiction (Ma et al., 2024; Wise and Robble, 2020). Neuromelanin-sensitive magnetic resonance imaging (NM-MRI) has shown a promising non-invasive technique for detecting catecholamine differences in the SN/VTA and LC associated with neuronal diseases and disorders (Fig. 1) (Sasaki et al., 2010). Neuromelanin is a byproduct of catecholamine metabolism, particularly in dopamine and noradrenaline synthesis and gradually accumulates in neurons as iron-neuromelanin complexes through oxidation and polymerization processes (Thaler and Politis, 2018; Jiang et al., 2022). While NM-MRI is sensitive to the presence of neuromelanin, the observed contrast may also be influenced by other tissue properties, such as water content or macromolecular composition in the SN and LC (Watanabe et al., 2014; Trujillo et al., 2017). Therefore, NM-MRI does not provide a direct measure of dopamine or noradrenaline levels but rather reflects neuromelanin concentration and potentially other tissue characteristics. It is important to realize that NM-MRI operates on a very different timescale than fMRI or PET and does not directly index real-time dopamine or norepinephrine signaling, nor does it capture phasic changes (Fig. 2, adapted from (Reneman et al., 2021)). Thus, this technique might offer indirect insights into long-term neurobiological changes associated with the dopaminergic and noradrenergic systems.

NM-MRI has been well-documented for investigating pathological changes in neurodegenerative conditions like Parkinson's disease (PD) (Sasaki et al., 2006; Hatano et al., 2017; Sulzer et al., 2018), but the application in psychiatric research remains limited. Studies recently demonstrate the potential of NM-MRI in detecting neuromelanin-related changes in psychiatric disorders (Lv et al., 2024; Wieland et al., 2021). Meta-analyses of NM-MRI in schizophrenia suggest its potential as a marker for dopaminergic dysfunction, though further research is needed (Wieland et al., 2021; Ueno et al., 2022; Trujillo et al., 2024). A systematic review additionally indicated the role of NM-MRI in mental disorders and found that it could serve as a proxy for dopaminergic and noradrenergic dysfunction (Lv et al., 2024). Wengler et al. (2025) recently provided a valuable overview of NM-MRI mechanisms and early clinical applications, establishing a methodological and biological foundation of NM-MRI.

The current review builds on these findings by exploring how NM-MRI aligns with both the dopaminergic and noradrenergic mechanisms of psychiatric disorders. It also evaluates its potential as candidate marker across a spectrum of psychiatric conditions. The existing literature on NM-MRI in psychiatric disorders will be discussed to guide future research efforts in applying NM-MRI in psychiatry.

## 2 Methods

This narrative review aimed to synthesize current evidence on NM-MRI in psychiatric disorders. This approach enabled integration of findings from both human and translational research, emphasizing conceptual and neurobiological trends. The literature search was conducted on April 14th^,^ 2025. Searches were performed in PubMed, Scopus, and ResearchGate databases. Search syntax was adapted to each database while preserving the same conceptual structure. Searches were performed in PubMed using a combination of MeSH terms and free-text fields, and in Scopus via Advanced Search using TITLE-ABS-KEY fields (see Appendix 1 for the full database search strategies). In ResearchGate, searches were conducted using combinations of similar keywords. No date limits were applied. Where available, filters for human studies and English-language publications were used.

Eligible studies included original research articles, systematic reviews, and meta-analyses published in peer-reviewed journals and written in English. Studies were included if they examined NM-MRI in relation to psychiatric populations or explored catecholaminergic mechanisms relevant to psychiatric pathophysiology. Abstract-only publications and preliminary results were excluded. To ensure completeness, additional articles were identified through backward and forward citation tracking of included studies. Eventually, twenty-six studies were included as described in Table 1.

**Table 1:** Study design of the 26 studies included in this review and their main findings.

Authors	Subject	System	ROI	NM-MRI outcome	Sample	Gender (M/F)	Mean age	Medication	MRI sequence	Main findings
Calarco et al., 2022	Late-life depression	NE	LC	CR	25 LLD - 23 HC	7/18 - 11/12	68.1 - 70.0	Antidepressant (n = 13)	2D GRE-MT	LC cr: No group differences; positive relationship between integrity of right rostral LC and cognitive performance; negative relationship between left caudal LC integrity and cognitive performance (Wilks' λ = 0.03, F(84, 162.44) = 1.66, p = <0.01)
Cassidy et al., 2019	Schizophrenia	DA	SN	CNR	33 SZ - 45 HC	23/10 - 27/18	33.9 - 34	Antipsychotic history (n = 17)	2D GRE-MT	SN cnr: No group differences; positive relationship between NM-MRI signal and psychosis severity in SZ (r = 0.38, p=0.044) and CHR (r=0.57, p=0.006)
Cassidy et al., 2020	Cocaine use disorder	DA	SN	CNR	20 CUD - 35 HC	20/0 - 35/0	47.3 - 45.1	NA	2D GRE-MT	SN cnr: ↑ CUD (ventral and lateral) (p < 0.05); No correlation in signal and SUD severity
Choi et al., 2023	Schizophrenia	DA	SN	CR	29 SZ - 63 HC	14/15 - 30/33	25.6 - 24.1	Antipsychotics (n = 29)	3D GRE-MT	SN cnr: ↑ SZ (t = −2.12, p=0.037); ↑ NM-MRI signal results in ↓ FST connectivity (β = −0.38, p = 0.042)
Guinea-Izquierdo et al., 2021	Late-life major depression disorder and amnestic mild cognitive impairment	NE	LC	CR	37 MDD - 21 aMCI - 31 HC	11/26 - 9/12 - 11/20	68.0 - 71.5 - 67.7	SSRIs (n = 6), SNRIs (n = 25) other (n=24)	2D FSE	LC cr: ↓ MDD compared to aMCI (δ = −0.53, p < 0.005) and HC (δ = −0.51, p=<0.001),specifically in MDD patients taking SNRIs (compared to HCs δ = 0.63, p < 0.001, and aMCI δ = 0.64, p < 0.001); no additive effect of MDD and aMCI
Jalles et al., 2020	First-episode Psychosis and substance abuse	DA	SN	CR	11 FEP - 19 S-FEP - 8 HC	NA	NA	NA	NA	internal SN cr: ↑ S-FEP compared to HC; external SN cr: ↑NS-FEP compared to S-FEP; LC cr: increasing trend in S-FEP
Jarcho et al., 2022	Substance abuse	DA	SN	CNR	33 SU	12/M-21/F	21.9	NA	2D GRE-MT	↓ SN cnr is associated with ↑ SA score b = −4.472, t(32) = −2.888, and p = 0.00073)
Kuai et al., 2024	Bipolar depression and Unipolar depression	DA	SN	CNR	46 BD - 38 UD - 42 HC	10/36 - 12/26 - 18/24	26.7 - 27.8 - 28.3	Medication naive	2D GRE-MT	SN cnr: ↓ BD and UD compared to HC(η2 = 0.195, p < 0.001)
Ma et al., 2024	Internet gaming disorder and tobacco use disorder	DA	SN	CNR	17 IGD - 14 TUD - 38 HC	17/0 - 13/1 - 38/0	20.9 - 22.5 - 20.8	NA	2D GRE-MT	SN cnr: ↑ IGD and TUD compared to HC (F=3.324,p=0.029; F=3.267, p=0.030); ↑ lateral SN cnr: ↑ IGD addiction severity (r = 0.50, p < 0.05), ↑ central SN cnr: ↑ TUD addiction severity (r = 0.52, p < 0.05)
McCall et al., 2024	Military Posttraumatic Stress Disorder	NE	LC	CNR	34 PTSD - 32 HC	26/8 - 19/13	47.5 - 47.1	Antidepressants (n = 20) SUD (n = 9)	2D GRE-MT	LC cnr: ↑ PTSD (t62=2.64, p=0.010); caudal LC signal positively correlated with hyperarousal symptoms (t56=2.70, p=0.040)and negatively correlated with depressive symptom severity (t26=−3.02, p=0.006)
Morris et al., 2022	Depression and anxiety disorders	DA	SN & VTA	Integrity	21 DAD - 22 HC	11/10 - 7/15	30.7 - 31.4	medication naive	3D GRE-MT	VTA integrity: ↓ in DAD (t = 1.71, p=0.047)and associated with lower extrinsic motivation (R = 0.407, p=0.041); SN integrity: no group differences
Morris et al., 2020b	Pathological anxiety	NE	LC	Volume	15 PA - 14 HC	6/9 - 10/4	38.7 - 39.9	medication naive	3D GRE-MT	LC volume: ↑ in PA (Cohen's d = 1.08, p = 0.024) is negatively associated with attentional (R=−0.505, FDR-corrected p = 0.020) and inhibitory control (R=−0.545, FDR-corrected p=0.015), and positively correlated with general distress (R=0.618, FDR-corrected p=0.006) anxious arousal (R=0.483, FDR-corrected p=0.021)
Pagliaccio et al., 2023	Obsessive-compulsive Disorder	DA	SN & VTA	CNR	64 OCD - 71 HC	23/41 - 27/44	11.3 - 10.4	ADHD medictions (n = 4), psychotropic free (n = 64), psychotropic naive (n = 71)	2D GRE-MT	SNcnr and VTAcnr: ↑ in OCD (Cohen's d = 0.51, p=0.004; Cohen's d = 0.50, p=0.006) and associated with lower lifetime symptom severity (t=−2.72, p=0.009) and a shorter illness duration (t=−2.22, p=0.03)
Perlman et al., 2024	Lifetime substance use	DA	SN/VTA	CNR	135 SU - 56 SUD	30/105 - 11/45	22.1	NA	2D GRE-MT	↑ SN/VTA cnr: ↑SU in women (B=0.55, p=0.02)
Sasaki et al., 2010	Depression and schizophrenia	DA/NE	SN & LC	CR	23 MDD - 23 SZ - 23 HC	12/11 - 15/8 - 10/13	49.4 - 44.9 - 47.0	Antipsychotics (n = 23), SSRI (n = 15), SNRI (n = 5), others (n = 33)	2D FSE	SN cr: ↑ SZ compared to MDD(p = 0.03), no differences between MDD and HC; LC cr: ↓ in MDD compared to Sz (p < 0.001), no differences between SZ and HC
Shibata et al., 2007	Depression	NE	LC	CR	20 MDD - 43 HC	11/9 - 21/22	49.1 - 49.4	Antidepressants (n = 20)	2D FSE	LC cr: ↓ MDD, specifically in rostral and middle LC (p=0.02 and p < 0.001)
Shibata et al., 2008	Depression and schizophrenia	DA/NE	SN & LC	CR	18 MDD - 20 SZ - 34 HC	10/8 - 13/7 - 17/17	44.6 - 44.6 - 43.8	Antipsychotics (n = 20, SSRI (n = 13), SNRI (n = 3), others (n = 24)	2D FSE	SN cr: ↑ SZ compared to MDD (p=0.025) and HC (p=0.023), no differences between MDD and HC; LC cr: ↓ in MDD compared to HC (p=0.003) and to SZ (p=0.035), no differences between SZ and HC
Slifstein et al., 2024	Schizophrenia	DA	SN/VTA	CNR	14 SZ - 12 HC	6/8 - 6/6	29.8 - 28.1	medication free (n = 9) medication naive (n = 4)	2D GRE-MT	SN/VTA cnr: No group differences; moderate associations between NM-MRI signal and anhedonia-related measures
Tavares et al., 2018	First-episode Psychosis and substance abuse	DA	SN	Volume	11 S-FEP -19 NS-FEP	6/5 - 15/4	23.0 -25.0	Antipsychotics (n = 30)	2D FSE	SN volume: ↑ S-FEP compared to HC (p=0.048)
van der Pluijm et al., 2024	Treatment resistant Schizophrenia	DA	SN/VTA	CR	47 RS - 15 NRS - 20 HC	32/15 - 11/4 - 14/6	24.1 - 21.3 - 22.7	Antipsychotics (n = 62)	2D GRE-MT	SN/VTA cr: ↑ RS compared to NRS and HC (F=4.1, p=0.02)
van Hooijdonk et al., 2023b	Schizophrenia	DA	SN	CNR	12 SZ - 16 HC	10/2 - 12/4	20.8 - 24.5	Antipsychotics (n = 12)	2D GRE-MT	SN cnr: No group differences; negative correlation between NM-MRI signal and dopamine synthesis capacity in HC (rho = -0.853, p < 0.001)
Vano et al., 2024	Schizophrenia	DA	SN/VTA	CNR	74 SZ - 80 HC	50/24 - 58/22	31.3 - 32.3	Antipsychotics (n = 57)	2D GRE-MT	SN/VTA cnr: ↑ SZ (effectsize=0.38, p=0.019)
Wang et al., 2021	Chronic cocaine use	DA/NE	SN/VTA & LC	CR	44 CU - 59 HC	37/7 - 44/15	46.5 - 43.7	NA	2D FSE	SN/VTA cr: No group differences; LC cr: ↑ in CU (p < 0.001)
Watanabe et al., 2014	Schizophrenia	DA/NE	SN & LC	CR	52 SZ - 52 HC	27/25 - 27/25	35.1 - 34.6	Antipsychotics (n = 52)	T2∗ 3D-spoiled GRE	SN cr: ↑ SZ (p < 0.01); LC cr: No group differences; in SZ < 30 years compared to HC difference was more prominent (p < 0.005)
Wengler et al., 2024	Antipsychotic-free Psychosis	DA	SN	CNR	42 SZ - 24 HC	25/17 - 12/12	34.3 - 35.9	Medication free (n = 42)	2D GRE-MT	SN cnr: ↑ in SZ (p=0.03); ↑SN cn: ↑ symptom severity (mean r=0.305, t37=2.24 p=0.01) and ↓ illness duration (t34=−2.28, p=0.03)
Yamashita et al., 2016	Schizophrenia	DA	SN & VTA & SN/VTA	CNR	14 SZ - 22 HC	11/3 - 14/8	37.0 - 40.0	Neuroleptics (n = 14)	T1 3D spoiled GRE	SN cnr: No group differences; SN/VTA cnr: ↓ in SZ (p=0.010); VTA cnr: ↑ in SZ (p=0.010); negative correlation with VTA cnr: in positive symptom (r=−0.69, p=0.012)

aMCI=amnestic mild cognitive impairment; BD=bipolar depression; CHR=clinical high risk; CNR=contrast; to; noise ratio; CR=contrast ratio; CUD=cocaine use disorder; CU=chronic cocaine use; DAD=depression and anxiety disorders; DA=dopamine; FEP=first; episode psychosis; FST=fronto; striatal thalamic circuit; HC=healthy controls; IGD=internet gaming disorder; LC=locus coeruleus; LLD=late; life depression; MDD=major depressive disorder; NA=not applicable; NE=norepinephrine; NRS=non; responding schizophrenic; NS; FEP=no substance abuse first; episode psychosis; OCD=obsessive; compulsive disorder; PA=pathological anxiety; PTSD=posttraumatic stress disorder; RS=responding schizophrenic; SA=substance abuse; SN=substantia nigra; SU=substance use; S; FEP=substance abuse first; episode psychosis; SZ=schizophrenia; TUD=tobacco use disorder; UD=unipolar depression; VTA=ventral tegmental area.

## 3 Catecholamine systems and NM-MRI

**Fig. 3:**
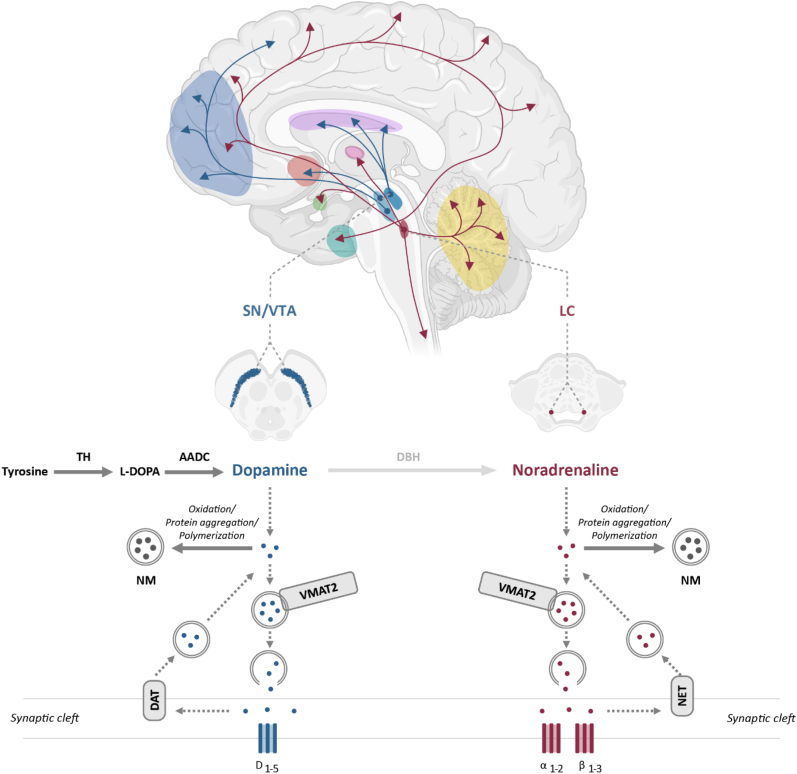
Illustration of the major dopaminergic (blue) and noradrenergic (burgundy) pathways in the brain. The dopaminergic system is shown with the dopaminergic cell bodies located in the substantia nigra (SN, in blue) and the ventral tegmental area (VTA, in blue), both residing in the brainstem. The dopaminergic dendrites of the nigrostriatal pathway project from the SN to the striatum (purple), while the mesocortical and mesolimbic pathways originate from the VTA and project to the prefrontal cortex (dark blue) and nucleus accumbens (red), respectively. The noradrenergic pathway, originating from the locus coeruleus (LC, in burgundy) in the brainstem, has widespread projections throughout the brain, including the prefrontal cortex (dark blue), hippocampus (turquoise), thalamus (pink), cerebellum (yellow), and spinal cord. In SN and VTA, dopamine is synthesized with neuromelanin as a byproduct. Tyrosine hydroxylase (TH) converts tyrosine to levodopa (L-DOPA), which is then converted to dopamine by aromatic acid decarboxylase (AADC). Dopamine is transported and stored in vesicles by vesicular monoamine transporter 2 (VMAT-2). Upon neuronal activation, dopamine is released into the synaptic cleft, where it binds to dopaminergic (D_1_–D_5_) receptors. Dopamine is cleared from the synaptic cleft by dopamine transporter (DAT). Excess cytosolic dopamine is converted to neuromelanin (NM). In the LC, noradrenaline is synthesized via a similar pathway, but with dopamine being converted to noradrenaline by dopamine β-hydroxylase (DBH). Noradrenaline is stored by VMAT-2 and released into the synaptic cleft, where it acts on adrenergic (α/β) receptors. Noradrenaline reuptake is performed by noradrenaline transporter (NET), and excess cytosolic noradrenaline is converted to neuromelanin. *Created using images from BioRender.*

Dopamine and noradrenaline are crucial neurotransmitters in the central nervous system, playing key roles in reward processing, motor control, arousal, mood regulation and cognitive function (Klein et al., 2019). The synthesis of dopamine starts with the enzyme tyrosine hydroxylase (TH) converting the amino acid tyrosine to levodopa (L-DOPA). This process requires tetrahydrobiopterin, oxygen, and iron as cofactors. Subsequently, L-DOPA is then converted into dopamine by the enzyme aromatic acid decarboxylase (AADC). Once synthesized, dopamine is transported into synaptic vesicles for storage by the vesicular monoamine transporter 2 (VMAT2). When stimulated, the vesicles undergo exocytosis, releasing dopamine into the synaptic cleft, where it can bind to dopaminergic receptors (D_1_-D_5_). D_1_-like receptors typically promote excitatory signaling through cyclic adenosine monophosphate (cAMP) activation, while D_2_-like receptors primarily mediate inhibitory effects by decreasing cAMP levels. After being released into the synaptic cleft, dopamine can either act on the postsynaptic neurons or be transported back into the presynaptic neuron by the dopamine transporter (DAT) (van Hooijdonk et al., 2023a). Cytosolic dopamine is either rapidly broken down or undergoes oxidative processes, leading to the formation of neuromelanin, see Fig. 3 for a detailed overview.

The synthesis of noradrenaline starts with dopamine, which is converted to noradrenaline by dopamine β-hydroxylase (DBH) in synaptic vesicles (Fig. 3) (Klein et al., 2019). Once released, noradrenaline binds to adrenergic receptors (α1, α2, β1, β2, and β3), mediating a range of physiological responses. Afterward, noradrenaline is reabsorbed into the presynaptic neuron by the noradrenaline transporter (NET), where it is either broken down or converted into neuromelanin (Fig. 3). Hence, neuromelanin, as a byproduct of the metabolism of dopamine and noradrenaline, might serve as a long-term marker of dopamine and noradrenaline activity within neurons (Sulzer et al., 2008). Alterations at different stages of the dopamine and noradrenaline synthesis and metabolism are associated with specific disruptions in several psychiatric disorders (Goddard et al., 2010; Suárez-Pereira et al., 2022).

Dopaminergic neurons in the midbrain, primarily located in the VTA and SN project to different brain regions via distinct pathways (Fig. 3) (Boyle et al., 2023). The SN is central to the nigrostriatal pathway, which projects to the striatum and plays a key role in behavioral regulation, learning and movement (i.e. habit formation, goal-directed motor responses) (Haber, 2014). The VTA on the other hand, projects via the mesolimbic and mesocortical pathways. The mesolimbic pathway, connecting the VTA to the nucleus accumbens, is involved in reward sensitivity (“liking”) and motivation (“wanting”) (Luo and Huang, 2016). The mesocortical pathway, projecting to the prefrontal cortex, regulates learning, reward sensitivity and emotion (Boyle et al., 2023). All these processes are crucial for healthy and pathological functioning.

Noradrenergic neurons are predominantly located in the LC, a small nucleus in the brainstem. The LC projects widely throughout the brain, including to the cortex, hippocampus, amygdala, and hypothalamus (Maness et al., 2022). Noradrenergic pathways are particularly important in modulating the fight-or-flight response and noradrenaline release during states of stress. This enhances alertness and redirects body resources to essential survival functions (Suárez-Pereira et al., 2022).

Respectively, NM-MRI captures neuromelanin-related contrast in the SN/VTA and LC, offering an indirect measure of dopaminergic and noradrenergic pathway integrity. Although the signal may be influenced by additional tissue properties, NM-MRI provides a practical approach for studying these catecholaminergic systems in humans (Sasaki et al., 2006).

## 4 Dopamine related disorders

Dopaminergic dysfunction has been implicated as a key neurobiological mechanism in several psychiatric disorders. Alterations in dopamine synthesis, release, and receptor signaling are thought to underlie a range of symptoms, from psychosis and mood disturbances to compulsive behaviors and addiction. In the following sections, we first examine disorders in which dopaminergic dysfunction plays a central role, summarizing current evidence from NM-MRI studies.

### 4.1 Schizophrenia spectrum disorders

The most prevalent investigated mental disorders using NM-MRI is schizophrenia. This psychiatric disorder is characterized by positive symptoms like delusions, disorganized speech, hallucinations, and negative symptoms like social withdrawal, flat affect and deficits in cognitive function (McCutcheon et al., 2020). Schizophrenia can be linked to dysregulation of dopamine neurotransmission (Stone et al., 2009; Howes and Kapur, 2009; Howes et al., 2013; Jauhar et al., 2017)Hyperdopaminergic activity in the mesolimbic pathway, manifested as increased dopamine synthesis capacity and dopamine release, is thought to drive positive symptoms, whereas hypodopaminergic activity in the mesocortical pathway has been implicated in negative symptoms (Howes and Kapur, 2009; van Hooijdonk et al., 2023a).

Several studies using post-mortem analyses and molecular imaging techniques, such as PET and SPECT, have detected altered dopaminergic function in individuals with schizophrenia. These findings consistently demonstrate increased presynaptic dopamine synthesis capacity and heightened amphetamine-induced dopamine release in patients with schizophrenia, whereas individuals at clinical high risk for psychosis show elevated dopamine synthesis capacity, particularly among those who later transition to psychosis (Howes et al., 2013; Jauhar et al., 2017; Kapur et al., 2000). Moreover, antipsychotic medication is found to impact the dopaminergic system, for example D_2_/D_3_ receptor occupancy is elevated following antipsychotic treatment (Howes et al., 2011, 2013; Stone et al., 2009; Jauhar et al., 2017; Kim et al., 2017; Tseng et al., 2018). Taken together, substantial evidence supports dysregulated presynaptic dopaminergic synthesis as a key mechanism in schizophrenia pathophysiology (Howes and Kapur, 2009).

Building on these findings, NM-MRI studies have evaluated dopaminergic functioning in schizophrenia. Some studies report increased NM-MRI signal in the SN of patients compared to healthy controls (Sasaki et al., 2010; Shibata et al., 2008; Watanabe et al., 2014; Choi et al., 2023; Vano et al., 2024). However, other studies found no difference of the NM-MRI signal in the SN of patients with schizophrenia compared to healthy controls (Cassidy et al., 2019; Slifstein et al., 2024; van Hooijdonk et al., 2023b; Wengler et al., 2024; Yamashita et al., 2016). Nonetheless, Cassidy et al. (2019) did find a higher NM-MRI signal to be associated with severity of psychosis in schizophrenic patients and clinical high-risk individuals. However, no differences were seen in mean NM-MRI signal between clinical high risk individuals, schizophrenia patients, and healthy controls. A recent study by Wengler et al. (2024) confirmed the positive correlation between NM-MRI signal and symptom severity in antipsychotic-free schizophrenia, but failed to replicate the association between psychosis-risk syndromes and NM-MRI signal in clinical high risk individuals. They also observed a negative association between NM-MRI signal and illness duration, which they associate with the dopamine sensitization theory. This theory states that dopaminergic neurons might degenerate or lose function because of hyperactivity (Wengler et al., 2024). In contrast, Slifstein et al. (2024) found moderate associations between NM-MRI signal and anhedonia-related measures in schizophrenia. These findings suggest that NM-MRI may be more sensitive to chronic dopaminergic dysfunction associated with anhedonia than to more short-term localized changes associated with psychosis. They attribute the differences in their findings to small sample size. Finally, Yamashita et al. (2016) analyzed the VTA separately from the SN, and found a significant decrease in NM-MRI signal in patients with schizophrenia compared to healthy controls. They proposed that hypoactivity in the VTA could be accompanied by compensatory hyperactivity in the SN, aligning with the dopamine hypothesis regarding negative and positive symptoms (Maia and Frank, 2017). However, they did not observe a significant increase in NM-MRI signal in the SN as a proxy of these compensatory mechanisms (Yamashita et al., 2016). Despite these mixed findings at the individual study level, two recent meta-analyses integrating these studies have concluded that NM-MRI signal in the SN is significantly increased in patients with schizophrenia compared to healthy controls (Wieland et al., 2021; Ueno et al., 2022). This suggests that NM-MRI may capture psychosis related changes, potentially even reflecting psychosis severity.

Van Hooijdonk et al. (van Hooijdonk et al., 2023b) investigated the relationship between NM-MRI signal in the SN and striatal dopamine synthesis capacity, measured in both healthy controls and patients with schizophrenia using [^18^F]F-DOPA PET. Contrary to their hypothesis, they found a significant negative correlation between NM-MRI signal and dopamine synthesis capacity in healthy controls. They suggested that higher neuromelanin levels may reflect lower cytosolic dopamine available for synthesis, potentially due to VMAT2 functioning. This correlation was absent in patients with schizophrenia, potentially due to factors such as symptom severity, the effects of antipsychotic medication, or the study's small sample size (van Hooijdonk et al., 2023b). A more recent study with a larger sample of 40 individuals with schizophrenia reported a correlation between NM-MRI signal in the SN-VTA and dopamine synthesis capacity in both the striatum and SN-VTA (Vano et al., 2024). In addition, Cassidy et al. (2019) used a multimodal approach combining NM-MRI with [^11^C]raclopride PET and fMRI. Their findings show that NM-MRI signal in the SN was associated with both dopamine release in the dorsal striatum and resting cerebral blood flow in the SN. To investigate the potential connections between NM-MRI signal and brain circuitry in schizophrenia, Choi et al. (2023) examined the relationship between NM-MRI and functional connectivity in the fronto-striato-thalamic (FST) circuit. This circuit is influenced by the mesocortical and nigrostrial pathways, of which alterations have been reported in schizophrenia (Sabaroedin et al., 2023). They found that reduced connectivity in the FST circuit was correlated with higher NM-MRI signal (Choi et al., 2023). Collectively, these results demonstrate the potential of NM-MRI as a proxy marker of dopaminergic functioning in the nigrostriatal circuit. Additionally, NM-MRI signal in the SN has been found to differ between schizophrenia patients who respond to first-line antipsychotic treatment and those who do not. Responders exhibit elevated NM-MRI signals compared to both non-responders and healthy controls (van der Pluijm et al., 2024).

These findings are in line with previous PET and SPECT results, suggesting that NM-MRI may capture psychosis-related changes and has potential as a proxy marker of dopaminergic function and treatment response. However, the variability in findings underscores the need for further research into methodological differences and confounding factors (Kim et al., 2017).

### 4.2 Substance-related and addictive disorders

Addiction is a complex condition where behavior, initially serving to provide pleasure or reduce distress, becomes compulsive and difficult to control. It can be marked by two defining characteristics: the repeated inability to manage the behavior, and the persistence of the behavior despite its harmful consequences (Goodman, 2008). The role of dopamine in addiction has been extensively studied over the past 40 years (Solinas et al., 2019). During the initial binge/intoxication stage of addiction, the behavior triggers dopamine release in the ventral striatum, producing a response directed at wanting to repeat the experience. As behavior becomes habitual, dopaminergic activity shifts from the ventral to dorsal striatum, which is associated with habit formation (Wise and Jordan, 2021; Volkow and Morales, 2015). In contrast, the withdrawal stage is marked by negative affect and hypoactivity of the dopaminergic receptors leading to negative reinforcement (Koob and Volkow, 2016). Additionally, during the craving/relapse phase, there is an activation of cortical areas and dopamine release in regions linked to emotions and memory (Volkow and Morales, 2015; Garrison and Potenza, 2014; Le Foll et al., 2022; Martinez et al., 2011; Volkow et al., 2019). Dysfunction of the dopaminergic system in substance and behavioral addiction has been investigated using various neuroimaging techniques, such as PET, SPECT, and fMRI, as well as post-mortem analyses. The majority of the studies indicate a reduction in D_2_ receptor expression and presynaptic dopamine release (Ashok et al., 2017a; Garrison and Potenza, 2014; Le Foll et al., 2022; Martinez et al., 2011; Volkow et al., 2019; Yuan et al., 2011).

Alterations in dopamine function in addiction have also been investigated using NM-MRI, with varying results. In contrast to the previous neuroimaging studies and post-mortem observations (Ahrens et al., 2024), most studies using NM-MRI found an increased signal in the SN, SN/VTA and lateral brain regions in substance use disorder, internet gaming disorder and tobacco use disorder where a decreased signal would be expected (Ma et al., 2024; Jalles et al., 2020; Cassidy et al., 2020; Tavares et al., 2018; Perlman et al., 2024). Perlman et al. (2024) demonstrated a significant positive association between cumulative substance use and NM-MRI signal in young women. They propose that the elevated neuromelanin accumulation reflects persistent dopamine alterations caused by prolonged substance exposure. Studies have demonstrated an increased NM-MRI signal (Jalles et al., 2020; Cassidy et al., 2020) and volume (Tavares et al., 2018) in individuals with a history of substance use, including first-episode psychosis patients who had used illicit substances and individuals diagnosed with substance use disorder. However, they did not find a significant correlation between the NM-MRI signal and addiction severity. Similarly, Ma et al. (2024) observed an increase in NM-MRI signal in internet gaming disorder and tobacco use disorder relative to healthy controls, although no significant correlation was found with addiction severities. Nevertheless, the positive directionality and magnitude of the effects they observed suggest a need for further research.

Cassidy et al. (2020) hypothesized that the dissimilarities between PET and NM-MRI studies could be explained by the redistribution of dopamine between vesicular and cytosolic pools during the dopamine synthesis cycle. In addicted individuals, PET studies using the [^18^F]DOPA PET radiotracer, which likely accumulates in synaptic vesicles, show decreased dopamine levels (Wu et al., 1997; Kumakura and Cumming, 2009). Whereas NM-MRI detects higher signal levels in addiction, reflecting elevated cytosolic dopamine concentrations (Sulzer et al., 2000; Zucca et al., 2014). This pattern aligns with evidence of reduced VMAT2 expression in cocaine users (Howell and Kimmel, 2008; Little et al., 2003). When VMAT2 expression is blunted, vesicular dopamine storage is reduced, leading to decreased presynaptic release. The resulting increase in cytosolic dopamine is prone to oxidation, ultimately contributing to neuromelanin formation. These findings potentially explain both the PET and NM-MRI results, which has also been discussed in relationship to schizophrenia by van Hooijdonk et al. (van Hooijdonk et al., 2023b). Another hypothesis that could explain these discrepancies is that elevated neuromelanin may reflect long-term accumulation of dopamine from episodic surges during addiction, which could persist beyond immediate PET measurements (Zucca et al., 2014). Since neuromelanin is only cleared following neuronal cell death, it serves as a long-term indicator of dopamine levels. This same hypothesis is proposed by Jarcho et al. (2022) which, in contrast to the other observations, found a negative correlation between substance use and NM-MRI signal in adolescents. They assign their deviated results to the shorter duration of substance use and fewer substance-related problems in their sample (Jarcho et al., 2022). Another possible hypothesis is that early-stage addiction is characterized by a hypodopaminergic state. In this state, reduced dopamine activity decreases responsiveness to natural rewards and increases the salience of drug-related cues, promoting risk-taking behavior. As addiction progresses, repeated substance use may sensitize the dopamine system, leading to increased dopamine release in response to drug cues and reinforcing cravings (Leyton and Vezina, 2014). This transition from hypodopaminergia to hyperdopaminergia could explain the different NM-MRI patterns in adolescents with shorter substance use compared to individuals with long-term addiction, as their dopamine systems are still in the early stages of sensitization. To achieve a more comprehensive understanding of dopamine system dysfunction in addictive disorders, future research should integrate NM-MRI with established molecular imaging techniques such as PET and SPECT to directly assess receptor availability and dopamine release. This multimodal approach may help clarify the specific contributions and limitations of each method in characterizing dopaminergic alterations in addiction.

### 4.3 Bipolar disorder

Bipolar disorder is a severe mood disorder characterized by alternating periods of mania and depression (Judd et al., 2008). The dopamine hypothesis of bipolar disorder is a primary theory explaining its pathopysiology, proposing that manic episodes result from hyperdopaminergia, while depressive phases arise from hypodopaminergia (Ashok et al., 2017b). Research supports a model in which increased striatal D_2_/D_3_ receptor availability enhances dopamine signaling, which may contribute to the emergence of manic symptoms. Whereas increased DAT levels is thought to reduce dopaminergic activity, potentially leading to depressive symptoms. This imbalance in dopamine receptor and transporter regulation may play a fundamental role in the cyclical nature of bipolar disorder (Ashok et al., 2017b). Supporting this, D_2_/D_3_ partial agonist have shown efficacy in treatment of bipolar disorder (Stahl et al., 2020). This is likely due to their ability to stabilize receptor activity by enhancing signaling when dopamine levels are low and reducing signal when dopamine levels are high, indicative of the manic and depressive states (Stahl, 2016). Neuroimaging studies have provided some insights into the dopamine dysfunction in bipolar disorder. Functional MRI studies have reported reduced activation in reward-related systems during depressive episodes (Redlich et al., 2015), and increased activity in dopaminergic reward circuits during manic episodes (Singh et al., 2013). However, findings in neuroimaging on bipolar disorder are inconsistent. Some studies using SPECT have observed increased DAT binding in depressive or euthymic bipolar disorder patients compared to healthy controls in bipolar disorder-II (Amsterdam and Newberg, 2007; Chang et al., 2010). While others using PET found a significant decrease of striatal DAT in depressive or euthymic bipolar disorder patients with bipolar disorder-I (Anand et al., 2011). These opposing results could be addressed by the different subtype of bipolar disorder studied. Supporting the initial theory, Jauhar et al. (2017) established that a higher [^18^F]DOPA PET radiotracer signal was correlated with positive symptoms in bipolar disorder.

One study has used NM-MRI to differentiate depressive bipolar disorder-II from unipolar depression (Kuai et al., 2024). In support of previous neuroimaging findings, both bipolar disorder-II depressive and unipolar depression patients showed reduced NM-MRI signal in the SN compared to healthy controls. Interestingly, bipolar disorder depressive patients exhibited higher SN volume measured by NM-MRI in comparison to unipolar depression patients, highlighting the potential utility of NM-MRI as a candidate marker to differentiate between these disorders (Kuai et al., 2024). Moreover, Morris et al. (2022) examined VTA dopaminergic cell integrity in individuals with depression and anxiety disorders, measured by NM-MRI signal intensity. They found that VTA integrity was reduced in those with mood/anxiety disorders.

Hence, although NM-MRI data in bipolar disorder are currently sparse, the initial findings are consistent with the dopamine hypothesis of bipolar disorder, which links depressive phases to hypoactivity of dopamine (Redlich et al., 2015; Kuai et al., 2024). It is important to note that the manic phases of bipolar disorder have not yet been examined with NM-MRI. Previous neuroimaging studies using PET and SPECT have reported changes in dopamine transporter and receptor availability across different mood states. However, these results have been inconsistent, especially regarding dopaminergic alterations during mania. An important direction for future research would be to utilize NM-MRI to specifically investigate dopaminergic hyperactivity during manic episodes. Longitudinal studies that follow patients across different mood states would be particularly valuable to determine whether NM-MRI can capture state-dependent changes in dopamine function in bipolar disorder, where NM-MRI can function as a reflection of the long-term dopaminergic activity, rather than acute fluctuations.

### 4.4 Obsessive compulsive disorder

Obsessive compulsive disorder (OCD) is a psychiatric condition characterized by intrusive, recurrent, and persistent unwanted thoughts (obsessions), and compulsions, such as repetitive behaviors or mental acts performed to relieve the anxiety triggered by the obsessions (American Psychiatric Association, 2022). Genetic, neuroimaging, and pharmacological studies have highlighted altered dopaminergic functioning in adults with OCD, including striatal dopaminergic hyperactivity. This hyperactivity is observed through imaging of DAT or dopamine receptor binding (Perani et al., 2008; Olver et al., 2009, 2010; Schneier et al., 2008; Koo et al., 2010). PET studies have shown significantly reduced D_1_ or D_2_ receptor binding and DAT availability in patients with OCD, which is thought to result from increased dopamine availability (Perani et al., 2008; Olver et al., 2009, 2010). Reduced receptor binding might be due to receptor downregulation or saturation, where receptors become less sensitive, reduced in number or saturated as a consequence of excessive dopamine levels. Whereas reduced DAT availability might suggest an increased dopamine concentration in the presynaptic cleft due to impaired reuptake or a compensatory mechanism to maintain dopaminergic signaling (Hesse et al., 2005). In support of the hyperactive dopamine theory, additional studies have provided evidence finding dopamine agonists and DAT inhibitors to induce or worsen OCD-like behaviors (Kelley et al., 2012) and correlating ventral striatal dopamine release to OCD-like behaviors (Wood and Ahmari, 2015; Denys et al., 2013).

A recent NM-MRI study in children with OCD revealed a significant increase in NM-MRI signal in the SN and VTA compared to healthy children (Pagliaccio et al., 2023). Higher NM-MRI signals were associated with lower lifetime symptom severity and shorter illness duration, hypothesized to reflect early compensatory dopamine activity in response to dysfunction such as reduced DAT availability. However, as the illness progresses and becomes more chronic, lower NM-MRI signals are found indicating reduced dopamine function. This pattern suggests that dopamine function changes over the course of the illness, with a shift from hyperactivity in early stages to a possible decline in function later in the disease (Pagliaccio et al., 2023).

These findings are consistent with PET and SPECT studies in adults and propose dopaminergic hyperactivity. However, the NM-MRI results also suggest that dopamine function can vary across different stages of the illness and between children and adults with OCD. Future research can focus on tracking NM-MRI signal across different illness stages and in relation to symptom progression, as well as examining the effects of commonly used medications. Such investigations would underscore the potential utility of NM-MRI as a non-invasive biomarker for disease monitoring throughout different phases, particularly in pediatric populations where conventional imaging modalities such as PET or SPECT are not feasible.

## 5 Noradrenaline related disorders

While dopaminergic dysfunction is central to several psychiatric disorders, the noradrenergic system also plays a pivotal role in mood and trauma-related conditions. Noradrenaline is essential for regulating arousal, attention, stress responses, and emotional processing (Suárez-Pereira et al., 2022). In the following sections, disorders in which noradrenergic dysfunction is prominent are examined. Current findings from NM-MRI studies that shed light on the involvement of this neurotransmitter system in psychiatric illness are highlighted.

### 5.1 Depressive disorders

Depressive disorders, including major depressive disorder (MDD), unipolar depression and late-life depression, are a leading cause for global disability, affecting around 300 million people (Nagy et al., 2020). It is characterized by physical symptoms like fatigue, weight loss, appetite changes. Emotional and cognitive symptoms include guilt, lack of motivation, sleep disturbances, and cognitive difficulties, with anhedonia, the inability to feel pleasure, being a central feature (Rice et al., 2019). The complexity of the pathological mechanism of depression makes effective pharmacological treatments challenging. Several theories have been proposed to explain its pathogenesis, one of which is the monoamine hypothesis (Cui et al., 2024). This theory proposes that clinical depression is driven by deficiencies in monoamine neurotransmitters like dopamine and noradrenaline (Cui et al., 2024). It is found that serotonin/noradrenaline reuptake inhibitors (SNRIs) increase the bioavailability of noradrenaline and are widely used as antidepressants in MDD patients (Cui et al., 2024; Luo et al., 2020). Several studies have used PET in the assessment of the noradrenergic system in depression and observed increased NET availability in depressive patients compared to healthy controls, leading to lower noradrenaline levels in the synaptic cleft (Moriguchi et al., 2017; Takano et al., 2014; Landau et al., 2023).

These abnormalities in the LC-noradrenergic system have also been demonstrated using NM-MRI. Studies showed that the NM-MRI signal in the LC was significantly lower in depressive patients compared to healthy controls (Sasaki et al., 2010; Shibata et al., 2007, 2008; Guinea-Izquierdo et al., 2021). Shibata et al. (2007) specifically revealed a decrease in NM-MRI signal in the rostral and middle parts of the LC. The rostral portion of the LC is thought to project mainly to the cerebral cortices and hypothalamus. These brain regions are specific for the ascending noradrenergic system that influences attention, arousal, mood and other higher cognitive processes (Fig. 3) (Loughlin et al., 1986). Moreover, Shibata et al. (2008) and Sasaki et al. (2010) established NM-MRI to be a useful marker for distinguishing schizophrenia, depression and healthy controls. When assessing the SN and LC together, depression was specifically associated with reduced NM-MRI signal in the LC. Furthermore, Guinea-Izquierdo et al. (2021) assessed the LC integrity, which captures signal intensity on NM-MRI. Apart from observing lower LC signal in MDD compared to healthy controls, they noticed that lower LC signal was specifically seen in patients taking SNRIs. They propose that this might result from SNRIs inhibiting noradrenaline reuptake, leading to a reduction in intracellular noradrenaline synthesis. This decrease in cytosolic noradrenaline could result in reduced neuromelanin in the LC (Guinea-Izquierdo et al., 2021). Nonetheless, one study found conflicting results, where the LC integrity signal did not show differences between late-life depression and healthy controls (Calarco et al., 2022). They did detect a strong relationship between LC integrity and cognitive performance, supporting earlier findings suggesting the LC is essential for cognitive functioning in healthy older adults (Hämmerer et al., 2018; Liu et al., 2020; Dahl et al., 2019). The discrepancies in results can be explained by differences in age. Shibata et al. (2008) included younger participants (<65 years old, mean of 44.6), Sasaki et al. (2010) spanned the entire adult range (22 to 83 years, mean of 49.1 years), and Calarco et al. (2022) studied late-life depression patients (mean age of 68 years). These results suggest that differences may be less noticeable in late life due to age-related decline or increased intraindividual variability (Sasaki et al., 2006; Liu et al., 2020). Moreover, Guinea-Izquierdo et al. (2021) reported a specific low LC signal in SNRIs users influencing the overall LC signal in the MDD group, while Calarco et al. (2022) included a low number of participants using SNRIs, partially explaining why no similar pattern was found.

The NM-MRI abnormalities in LC integrity and noradrenergic signaling are in line with the monoamine hypothesis of lower noradrenaline levels in depressive disorders. In addition, the controversy in the function of SNRIs and its noticeable effect in NM-MRI signal indicate a disruption in the LC-noradrenaline system. Further research into how SNRIs influence noradrenergic dynamics and their long-term effects in depression is needed.

### 5.2 Posttraumatic stress disorder

Posttraumatic stress disorder (PTSD) is recognized as a trauma-related disorder, which develops in individuals exposed to events involving a threat of death or serious injury (Hardy and Mueser, 2017). Individuals with PTSD often re-experience trauma through nightmares, flashbacks, and intrusive memories. Symptoms typically include negative mood changes, cognitive difficulties, heightened arousal, concentration issues, and avoidance behaviors (Cordova et al., 2017). Research has indicated the role of catecholamines, particularly noradrenaline, in the pathophysiology of PTSD (Strawn and Geracioti, 2008). Repeated acute traumatic stress elevates noradrenaline, consolidating long-lasting negative emotional memories. Reactivation of these memories’ trigger hyperarousal, which later shift to a "numbing" state due to noradrenergic burnout (Hendrickson and Raskind, 2016). The effectiveness of drugs like prazosin (α1 antagonist), clonidine (α2 agonist), and propranolol (β antagonist) in treating early PTSD symptoms like sleep disturbances and nightmares, highlight the role of a dysfunctional noradrenergic system in trauma-related syndromes (Hendrickson and Raskind, 2016; Morris et al., 2020a; Olff and van Zuiden, 2017; Ouyang et al., 2012). However, there is a notable lack of *in-vivo* studies examining noradrenergic regulation in PTSD.

Recent studies using NM-MRI have shown promise in assessing noradrenaline in PTSD (McCall et al., 2024; Morris et al., 2020b). McCall et al. (2024) reported increased NM-MRI signals in the LC in military PTSD and showed that the caudal LC signal was positively correlated with hyperarousal symptoms in PTSD while negatively correlated with the severity of depressive symptoms. This is consistent with previous findings where depression severity was also associated with a significantly lower caudal LC signal (Shibata et al., 2007). Furthermore, Morris et al. (2020b) examined the volume of the LC using NM-MRI in anxiety and stress-related disorders including PTSD. They observed a larger LC to be negatively associated with attentional and inhibitory control but positively correlated with anxious arousal.

These NM-MRI results support the "numbing" theory, where elevated LC signals may reflect heightened noradrenaline levels during traumatic memory reactivation, contributing to hyperarousal (Hendrickson and Raskind, 2016). Whereas reduced LC signal or volume observed in depression may indicate “noradrenergic burnout”, consistent with emotional numbing. However, larger studies are needed to clarify the relationship between NM-MRI signal in the LC and symptom dimensions such as hyperarousal and emotional numbing. Further research on how NM-MRI findings relate to noradrenergic system dysregulation observed in other imaging modalities can be explored.

### 5.3 Substance use disorder

While dopamine has been extensively studied in the context of substance use disorder, noradrenaline has also been implicated in the neuropathology of this disorder (Weinshenker and Schroeder, 2007). In humans, genetic polymorphisms in the NET gene have been shown to modulate mood responses to d-amphetamine, with these polymorphisms located at transcription factor binding sites, likely affecting NET expression (Dlugos et al., 2007). Although less is known about the effects of prolonged stimulant use on noradrenaline neurotransmission compared to the involvement of the dopamine system in addiction, a previous PET study demonstrated that NET is upregulated in humans addicted to cocaine, which would result in low noradrenaline levels in the synaptic cleft (Ding et al., 2010).

Wang et al. (2021) found and increased NM-MRI signal in the LC of patients with chronic cocaine exposure compared to healthy controls. Complementary, it was established that psychosis patients, particularly those with comorbid substance abuse, exhibit increased NM-MRI signal in the LC (Jalles et al., 2020). These findings confirm previous PET studies that observed NET upregulation, suggesting increased noradrenaline reabsorption and elevated cytosolic noradrenaline levels, which is more susceptible to oxidation and, consequently, neuromelanin formation.

## 6 Discussion

The current review examined the potential of NM-MRI as a non-invasive tool for visualizing the dopaminergic and noradrenergic system in several major psychiatric disorders. NM-MRI has shown promise in disorders where catecholaminergic dysfunction is implicated, particularly schizophrenia, addiction and depression (Table 1), but prospective studies are needed to establish its utility for diagnosis, monitoring, or predicting treatment outcomes.

### 6.1 Summary of current NM-MRI findings

In schizophrenia, most studies report increased NM-MRI signal in the SN, with some evidence linking NM-MRI signal to psychosis severity and treatment response (Sasaki et al., 2010; Wengler et al., 2025; Shibata et al., 2008; Watanabe et al., 2014; Choi et al., 2023; Vano et al., 2024; Cassidy et al., 2019). Similarly, substance use disorders and behavioral addictions are associated with increased NM-MRI signal in the SN, and there are indications of increased NM-MRI signal in the LC (Ma et al., 2024; Ahrens et al., 2024; Jalles et al., 2020; Cassidy et al., 2020; Tavares et al., 2018; Perlman et al., 2024; Wang et al., 2021). In contrast, depression studies consistently report decreased NM-MRI in the LC compared to healthy controls, aligning with the monoamine hypothesis of reduced noradrenergic function (Sasaki et al., 2010; Shibata et al., 2007, 2008; Guinea-Izquierdo et al., 2021). Initial NM-MRI findings in bipolar disorder, OCD, PTSD, and anxiety disorders are promising but the evidence is still limited (Kuai et al., 2024; Morris et al., 2020a, 2022; Pagliaccio et al., 2023; McCall et al., 2024). To advance the field, future research should prioritize larger, well-characterized cohorts and longitudinal studies to systematically examine NM-MRI signal across different clinical states, illness stages, and symptom dimensions. Such studies should also evaluate the influence of medication and comorbidities.

### 6.2 Synthesis of NM-MRI findings

The NM-MRI findings highlights both converging and diverging evidence between NM-MRI and other neuroimaging modalities. For example, PET/SPECT studies often report reduced presynaptic dopamine function in addiction. In contrast, NM-MRI findings suggest increased neuromelanin accumulation, which may reflect long-term cytosolic dopamine changes rather than acute synaptic activity. Similarly, correlations between NM-MRI and PET/SPECT markers of dopamine function are sometimes observed (Cassidy et al., 2019; Vano et al., 2024), but not always consistent (Cassidy et al., 2020; Wu et al., 1997; van Hooijdonk et al., 2023b; Zucca et al., 2014). Such discrepancies underscore the importance of interpreting NM-MRI as an indirect, cumulative marker of catecholaminergic metabolism, rather than a real-time measure of neurotransmitter release. It is important to note that many psychiatric disorders exhibit state-dependent changes in dopamine and noradrenaline, as shown by PET and SPECT studies (Redlich et al., 2015; Singh et al., 2013; Amsterdam and Newberg, 2007). For example in bipolar disorder, dopamine function differs between depressive and manic episodes (Ashok et al., 2017b). However, it remains unclear whether NM-MRI can detect such state-dependent changes, as existing NM-MRI studies have focused only on depressive states. On the other hand, in disorders such as OCD, schizophrenia, and substance use disorders, there is some evidence that NM-MRI can capture differences related to illness progression, distinguishing between early and late disease stages (Cassidy et al., 2019; Jarcho et al., 2022; Pagliaccio et al., 2023). These findings indicate that, although NM-MRI appears sensitive to long-term or cumulative changes, its capacity to reflect acute, state-dependent alterations requires further investigation. Medication effects are another important consideration, as they are known to influence PET/SPECT outcomes (Howes et al., 2011, 2013; Stone et al., 2009). Yet, findings with NM-MRI remain inconsistent. For example, SNRIs have been shown to decrease neuromelanin signal in the LC in depression (Guinea-Izquierdo et al., 2021), whereas antipsychotic treatment in psychosis did not change NM-MRI signal in the SN over six months (van der Pluijm et al., 2024) These inconsistencies may be due to differences in age, illness duration, medication status, or other clinical variables. Additionally, other potential influences such as comorbidity and sex remain underexplored and warrant further investigation.

### 6.3 Potential of NM-MRI in psychiatry

Despite these challenges, one of the most promising aspects of NM-MRI is its non-invasive nature without radiation exposure, making it suitable for longitudinal studies and for early diagnosis and monitoring. Unlike PET or SPECT, which is often unsuitable for children due to radiation risks, NM-MRI offers a safer method to study young individuals, critical since many psychiatric disorders begin in childhood or adolescence. Nevertheless, pediatric NM-MRI also presents important challenges such as motion artefacts, smaller brain structures, and ongoing neuromelanin development, all of which can complicate data acquisition and interpretation. Addressing these challenges will be essential, but the feasibility of NM-MRI in these populations highlights its potential and underscores the need for further research across a broader range of psychiatric disorders (Schneier et al., 2008; Olver et al., 2010; Landau et al., 2023; Weinshenker and Schroeder, 2007; Wang et al., 2021; Greydanus et al., 2021).

Disorders such as anxiety, anorexia nervosa, ADHD, autism spectrum disorder, and Tourette's syndrome also involve catecholaminergic alterations, highlighting the broader relevance of NM-MRI for neurobiological dysfunction across diverse clinical populations. Notably, anxiety has primarily been studied using NM-MRI in comorbid contexts, e.g. depression and PTSD, with LC integrity correlating with anxiety symptoms (Morris et al., 2020b, 2022). Given that repeated stress may contribute to LC dysregulation, which plays a role in maladaptive fear responses or anxiety disorders, further studies focusing exclusively on anxiety are needed (Morris et al., 2020a). As well as anorexia nervosa, where increased D_2_/D_3_ receptor binding was seen in recovered patients compared to healthy controls using PET, suggesting a dopamine-related disturbance (Frank et al., 2005). Currently, an ongoing study uses NM-MRI to assess midbrain neuromelanin and its relationship to illness duration, reward behaviors, and brain activity in reward systems in anorexia nervosa (Murray et al., 2024). Another disorder that is characterized by alterations in the dopaminergic and noradrenergic system is ADHD. Young adults with ADHD often exhibit increased DAT density, particularly in the midbrain and striatum (Krause et al., 2000; Cheon et al., 2003). Additionally, increased postsynaptic dopamine receptor density is shown to be linked to attention deficits (Lou et al., 2004). Methylphenidate (Ritalin), the most prescribed medication for ADHD, acts as a DAT antagonist (Greydanus et al., 2021; Schrantee et al., 2016). An alternative drug that blocks the NET has also been shown to improve clinical symptoms of ADHD (del Campo et al., 2011; Keehn et al., 2021). Given these catecholaminergic alterations, NM-MRI could be a valuable tool in investigating ADHD. Additionally, in the pathophysiology of autism spectrum disorder prior work has also hypothesized that the dopaminergic/noradrenergic systems may be implicated, where D_1_ receptor binding showed a negative correlation between specific autism symptoms in individuals with autism spectrum disorder (Kubota et al., 2020). Moreover, children with autism spectrum disorder show atypical increased tonic activation of the LC-noradrenaline system and is associated with poorer attentional disengagement (Keehn et al., 2021). NM-MRI may therefore offer a unique opportunity to examine dopaminergic and noradrenergic alterations in autism spectrum disorder. Finally, tics and compulsions seen in Tourette's syndrome may be linked to dopaminergic dysfunction, specifically decreased striatal D_2_/D_3_ receptor availability, which might reflect higher endogenous dopamine levels. A similar pattern has been reported in OCD, where NM-MRI has shown its potential (Denys et al., 2013; Steeves et al., 2010). To summarize, NM-MRI represents a promising tool for investigating dopamine and noradrenaline function, with potential applicability across diverse clinical populations, though further prospective and cross-diagnostic studies are required to establish its utility as a biomarker.

### 6.4 Biological and methodological considerations

While NM-MRI offers unique advantages, including its non-invasive nature and suitability for pediatric populations and longitudinal studie designs, key biological and methodological challenges must be addressed to fully realize its clinical utility. Biologically, the NM-MRI signal reflects the accumulation of neuromelanin, which is shaped by long-term catecholaminergic metabolism (Zucca et al., 2014). However, this signal can also be influenced by other tissue properties, such as water and iron content (Trujillo et al., 2017). As a result, NM-MRI does not provide a direct measure of dopamine or noradrenaline levels. Accordingly, caution is warranted in interpreting these findings, particularly when making cross-disorder comparisons, relating results to transient clinical states, or extrapolating from PET and SPECT data.

Methodologically, a major challenge is the wide variety of NM-MRI acquisition and analysis methods used across studies, see Box 1 for a brief overview. A comprehensive discussion of these methodological variations is provided in Wengler et al. (Wengler et al., 2025). Standardizing NM-MRI protocols and conducting more detailed investigation of subregional differences in NM-MRI signal, might improve reliability and specificity in clinical and research settings (van der Pluijm et al., 2021). Another issue to consider is that NM-MRI may be limited by its sensitivity to signal variations across different subregions of the SN and VTA since these are relatively small structures (Cassidy et al., 2019). However, employing a voxelwise NM-MRI approach may improve anatomical sensitivity and allow for finer subregional distinctions, which are particularly important given the small size of the SN, VTA, and LC. Currently, most studies employ a region of interest approach that considers these regions as a whole, potentially missing out on finer subregional differences that could provide more specific diagnostic or prognostic information (Cassidy et al., 2019).

Box 1 **Methodological heterogeneity in NM-MRI studies**

**Acquisition variations**.

NM-MRI acquisition differs across studies in magnet strength, sequence type, parameter choices, and scan orientation. These choices affect signal contrast, signal-to-noise ratio, partial volume effects, and sensitivity to specific nuclei. No single acquisition protocol has been universally adopted, contributing to heterogeneity in reported signal measures.

• **Field strength:** typically 3 T, some studies have used 7 T.
• **Sequence types:** 2D or 3D gradient echo versus turbo spin echo, with or without magnetization transfer (MT) pulses and differences in repetition/echo time, and flip angle.
• **Parameter choices:** slice thickness, field of view, MT strength, number of signal averages.
• **Scan orientation:** axial, oblique, or coronal planes, which can influence partial volume effects.

**Outcome measures**.

• **Contrast ratio:** Signal outcome metrics typically include *contrast ratio (CR)* and *contrast-to-noise ratio (CNR).* Different studies use different reference regions and formulas, meaning absolute CR/CNR values are not directly comparable across studies.
  o *CR* is calculated as the difference between mean NM-MRI signal in the target region (e.g., SN or LC) and a reference region, divided by the reference signal.
  o *CNR* additionally incorporates noise estimates (e.g., standard deviation or mode in the reference region) and may better reflect signal reliability.
• **Volume measures:** Some studies measure the volume of NM-rich nuclei (SN, LC) using intensity-thresholded or manual segmentation. Volume provides complementary information to signal intensity. Small nuclei and partial-volume effects make volume estimates sensitive to acquisition resolution and ROI definition.
• **Signal integrity:** In this approach, a MT-enhancement image is generated by dividing a MT weighted image by a non-MT image. This highlights voxels whose signal is influenced by large macromolecules such as neuromelanin.

**Analysis approaches**.

• **Region of interest (ROI):** Average signal across anatomically defined nuclei (e.g., SN/VTA or subareas), offering robustness but potentially obscuring spatial patterns. ROI delineation choices (manual, algorithmic, or atlas-based) influence measured outcomes, e.g. LC is very small and segmentation often relies on signal intensity thresholds or semi-automated methods.
• **Voxelwise analyses:** Assess signal variation at each voxel after normalization to standard space, increasing spatial specificity and reducing statistical circularity but requiring more intensive processing.

**Interpretation caution**.

Variability in acquisition, processing, and quantification means that consistency in the direction of effects across studies is often more informative than absolute signal magnitudes. Standardization efforts, including consensus on sequence parameters and analysis pipelines, are essential to improve comparability and enable replication across cohorts.

Alt-text: Box 1

Another important consideration is the impact of specific confounders, such as medication exposure, age, or sex on NM-MRI signal variability. However, data on these factors in psychiatric disorders remain limited. In psychosis, some studies have found that NM-MRI contrast correlates with clinical features specifically in antipsychotic-free cohorts (Wengler et al., 2024). However, meta-analyses including medicated and non-medicated psychosis cohorts have reported no consistent associations between NM-MRI signal and antipsychotic dose (Wieland et al., 2021; Ueno et al., 2022). Treatment response, rather than medication exposure itself, may be a more important moderator; first-episode non-responders show lower SN NM-MRI contrast than responders, while antipsychotic dose and duration do not appear to have a direct effect (van der Pluijm et al., 2024). Outside of psychosis, preliminary evidence suggests that medication may influence NM-MRI signal. For instance, in Parkinson's disease, higher L-DOPA doses are associated with lower SN NM-MRI signal (Hatano et al., 2017), and in depression, lower LC signal has been observed specifically in patients taking SNRIs (Guinea-Izquierdo et al., 2021). Although neuromelanin accumulates with normal aging in the SN and LC (Liu et al., 2020), age effects are generally absent or limited in the SN of psychosis cohorts (Wieland et al., 2021; Ueno et al., 2022; Wengler et al., 2024). Similarly, NM-MRI studies of the SN show no clear sex effects in psychosis (Wieland et al., 2021; Ueno et al., 2022), whereas some studies of the LC report differences in NM-MRI signal between healthy men and women (Riley et al., 2025; Clewett et al., 2016). Overall, these findings are limited and may be confounded by disease severity or other factors. Therefore, further research is needed to clarify the influence of medication, age, and sex on NM-MRI signal variability in psychiatric disorders.

A further challenge lies in the overlap of NM-MRI signal across various psychiatric disorders. Elevated NM-MRI signals in the SN and VTA have been observed in both schizophrenia and substance use disorders, raising concerns about whether NM-MRI can reliably differentiate between disorders or whether its value lies more in monitoring treatment response or disease progression within a single condition. At the same time, this overlap may reflect shared catecholaminergic mechanisms that transcend diagnostic boundaries, such as disruptions in reward processing, motivation, or stress regulation observed across schizophrenia, depression, and addiction (Solinas et al., 2019; Redlich et al., 2015; Morris et al., 2022). From this perspective, NM-MRI could serve as candidate transdiagnostic marker, capturing core neurobiological dysfunctions rather than disorder-specific alterations. Refining region-specific and neurotransmitter-specific analyses, such as distinguishing dopaminergic alterations in the SN/VTA from noradrenergic changes in the LC, may help enhance diagnostic specificity. Hence, it is important to investigate the differential diagnostic utility of NM-MRI more thoroughly.

### 6.5 Future directions

To enhance the differential diagnostic specificity of NM-MRI, future research should integrate NM-MRI data from both dopamine-rich regions, like the SN and VTA, and noradrenaline-rich regions, like the LC. This combined approach may help differentiate between disorders involving dysregulation of both neurotransmitters, such as addiction, ADHD, bipolar disorder, and depression (Howell and Kimmel, 2008; Kuai et al., 2024; Morris et al., 2020b, 2022; Keehn et al., 2021), as shown in studies differentiating schizophrenia and depression (Shibata et al., 2008; Watanabe et al., 2014). While NM-MRI has been used to study both noradrenaline function and dopaminergic function, most studies have focused on one system at a time. Future studies should prioritize combined analyses, as this could offer valuable insights into the interrelated roles of these neurotransmitter systems in psychiatry. For instance, interactions between noradrenaline and dopamine systems may influence emotional processing and cognitive function (Ranjbar-Slamloo and Fazlali, 2020).

Comparative studies across multiple psychiatric diagnoses, such as schizophrenia, depression, bipolar disorder, and anxiety, will be essential in determining whether this NM-MRI can truly distinguish between different conditions or is better suited as a transdiagnostic marker. It is important to recognize that dopamine and noradrenaline alterations do not strictly follow diagnostic classifications, with notable neurobiological heterogeneity within diagnoses. For instance, treatment resistant schizophrenia shows distinct neuromelanin profiles compared to treatment-response patients (van der Pluijm et al., 2024). Understanding therapeutically relevant neurobiological variation from a transdiagnostic perspective may be even more clinically meaningful, as this heterogeneity could guide more personalized and targeted treatment approaches.

Longitudinal studies could support this endeavor by examining NM-MRI signal over time in patients undergoing various treatments or during the natural course of psychiatric disorders. This would help establish NM-MRI as a potential tool for monitoring treatment response or predicting relapse and treatment resistance in patients (van der Pluijm et al., 2024). Additionally, future studies could investigate the ability of NM-MRI to identify and monitor individuals at high risk for developing psychiatric disorders (Cassidy et al., 2019; Wengler et al., 2024), potentially aiding in early diagnosis and intervention.

Large-scale, multi-site collaborations using harmonized NM-MRI protocols will be crucial for increasing reproducibility and statistical power. Including pediatric and adolescent cohorts will allow capturing early neurobiological changes, while task-based or pharmacological NM-MRI paradigms could help probe the functional relevance of catecholaminergic alterations. Combining NM-MRI with other modalities such as fMRI or PET may further help understand the relationship between catecholaminergic integrity and (network-level) brain function (van Hooijdonk et al., 2023b; Sabaroedin et al., 2023).

## 7 Conclusion

This review adds to an increasing body of work examining NM-MRI as an *in-vivo* marker in psychiatric disorders and paves the way toward future investigations. Based on the neurobiology of numerous psychiatric disorders, NM-MRI has potential as a non-invasive tool for studying the dopaminergic and noradrenergic systems across various psychiatric disorders. While further research is needed to overcome its limitations and expand its applications, NM-MRI may offer insights that could inform future efforts in diagnosis, treatment monitoring, and outcome prediction in psychiatry.

## Funding

This research did not receive any specific grant from funding agencies in the public, commercial, or not-for-profit sectors.

## Declaration of competing interest

The authors declare that they have no known competing financial interests or personal relationships that could have appeared to influence the work reported in this paper.

## Figures and Tables

**Table:** 

PubMed	Scopus
("neuromelanin"[Title/Abstract]	TITLE-ABS-KEY (neuromelanin
OR "neuromelanin-MRI"[Title/Abstract]	OR "neuromelanin MRI"
OR "neuromelanin-sensitive"[Title/Abstract]	OR "neuromelanin-sensitive"
OR "neuromelanin sensitive"[Title/Abstract]	OR "neuromelanin sensitive"
OR "NM-MRI"[Title/Abstract]	OR "neuromelanin contrast"
OR "neuromelanin imaging"[Title/Abstract]	OR "neuromelanin imaging"
OR "neuromelanin contrast"[Title/Abstract]	OR "NM-MRI"
OR (neuromelanin[Title/Abstract] AND MRI[Title/Abstract])	OR "neuromelanin magnetic resonance imaging")
OR "neuromelanin magnetic resonance imaging"[Title/Abstract])	AND
AND	TITLE-ABS-KEY ("psychiatr∗"
("psychiatr∗"[Title/Abstract]	OR "mental disorder∗"
OR "mental disorder∗"[Title/Abstract]	OR schizophrenia
OR schizophrenia[Title/Abstract]	OR psychosis
OR psychosis[Title/Abstract]	OR depression
OR depression[Title/Abstract]	OR "bipolar disorder"
OR "bipolar disorder"[Title/Abstract]	OR anxiety
OR anxiety[Title/Abstract]	OR PTSD
OR PTSD[Title/Abstract]	OR "post-traumatic stress disorder"
OR "post-traumatic stress disorder"[Title/Abstract]	OR OCD
OR OCD[Title/Abstract]	OR "obsessive-compulsive disorder"
OR "obsessive-compulsive disorder"[Title/Abstract]	OR addiction
OR addiction[Title/Abstract]	OR "substance use disorder"
OR "substance use disorder"[Title/Abstract]	OR autism
OR autism[Title/Abstract]	OR ADHD
OR ADHD[Title/Abstract]	OR "neurodevelopmental disorder∗")
OR "neurodevelopmental disorder∗"[Title/Abstract])	
